# Telocytes, exosomes, gap junctions and the cytoskeleton: the makings of a primitive nervous system?

**DOI:** 10.3389/fncel.2013.00278

**Published:** 2014-01-03

**Authors:** John Smythies, Lawrence Edelstein

**Affiliations:** ^1^Center for Brain and Cognition, University of CaliforniaSan Diego, CA, USA; ^2^Medimark CorporationDel Mar, CA, USA

**Keywords:** telocytes, exosomes, gap junctions, cytoskeleton, stem cells, homeostasis, signaling

## The facts

Telocytes (TCs) form a remarkable new cell species found in many types of tissue. They were discovered by Professor Laurentiu-Mircea Popescu of Roumania in 2005 (Popescu et al., 2005), and are characterized by having very small cell bodies (consisting of a nucleus and a small amount of cytoplasm) and “extremely long and thin” tubular processes called telopodes (up to 100 micrometers long, yet only 20–200 nanometers wide). Telopodes consist of long thin tubes (called podomers) interspersed with short dilations (called podoms) that have the appearance of axonal boutons en passage. Podoms contain abundant mitochondria, calveoli and endoplasmic reticulum (Cretoiu et al., 2012a, 2013). TCs form a dense convoluted network linking TCs with each other, and with many other cell types including secretory acini and exocrine epithelial ducts, nerve fibers, macrophages, and blood vessels (Nicolescu et al., 2012; Popescu et al., 2012; Bosco et al., 2013). Gherghiceanu and Popescu (2012) report that TCs make contact with virtually all types of cells in the human heart including Schwann cells, endothelial cells, pericytes, macrophages, mast cells, fibroblasts, stem cells/progenitors, and working cardiomyocytes. In the urinary system they establish numerous contacts *inter alia* with macrophages in the sub-capsular space of the kidney, or with smooth muscle cells, nerve endings as well as blood capillaries in the ureter and urinary bladder (Zheng et al., 2012). In the esophagus the cells that they make contact with include macrophages and lymphocytes (Chen et al., 2013). In the small intestine TCs encircle muscle bundles, nerve structures, blood vessels, fundi of gastric glands and intestinal crypts Vannucchi et al. (2013).

These networks contain embedded stem cells (Luesma et al., 2013). Connections by TCs with all of the aforementioned entities are made both by electron-dense nanocontacts involving junctions, and via exosomes (Nicolescu et al., 2012; Popescu et al., 2012; Luesma et al., 2013). Exosome release occurs along the telopodes (Cretoiu et al., 2012b). In the eye this gap-junction-linked network is prominent in the uvea and sclera. Moreover, exosomes (with a diameter up to 100 nm) are delivered by TCs to a wide variety of cells including cells of the iris stroma (Luesma et al., 2013), leading these authors to suggest that TCs may be involved in integrating neural, vascular and endocrine processes. Several authors have suggested that TCs are involved in tissue protection, remodeling and regeneration (Xiao et al., 2013; Zhao et al., 2013; Zheng et al., 2013) as well as mechanical support, spatial relationships with different cell types, intercellular signaling and modulation of intestinal motility (Cretoiu et al., 2013; Milia et al., 2013).

Their presence in the meninges and in the choroid plexus/subventricular zone within the proximity of neural stem cells led Popescu et al. (2012) to suggest that TCs may be involved in modulating neural stem cell fate. Ongoing investigations are revealing increasingly important roles for TCs in the stabilization of healthy tissue function, so as to minimize inflammatory tissue damage and possibly oxidative damage to particular organs (Haines et al., 2013). For example, Zhao et al. reported an inverse correlation between the magnitude of infracted zones of rat myocardium and cardiac TC density (Zhao et al., 2013). TCs, therefore, function in tandem with stem cells in anatomical locations designated “stem-cell niches” resulting in an enhancement of their engraftment capability (Polykandriotis et al., 2010). Ceafalan et al. (2012) have suggested that TCs are involved in skin homeostasis, remodeling, regeneration and repair.

It is of interest to note that, on a micro scale, a TC shares some of the properties of the claustrum (a nucleus in the mammalian telencephalon), namely, keeping its fingers in many pies, therein elevating the role and prospective benefits of gap junctions and a thin topology (Smythies et al., 2012, 2014). Both structures seem to be closely involved in integrating the function of multiple other structures.

## Explanations of the facts

TCs clearly form some type of intercellular signaling system, but what the exact nature of the signals are—and what their function is - remains obscure. Several authors have suggested that TCs “integrate functions or processes,” but details are lacking. It is the purpose of this paper to explore some possible answers to this question. Gap junctions can transmit ions and small molecules in either direction, but they are too small to transmit protein molecules, let alone exosomes. They also transmit electrical signals [see Nielsen et al. (2012) for a comprehensive review]. Recently the passage of siRNAs and synthetic oligonucleotides of approximately 2–4 kDa has been reported (Valiunas et al., 2005). Moreover, it has been shown that TCs express a broad range of microRNAs, such as pro-angiogenic and stromal-specific miRs (Zheng et al., 2013). In particular, miRNA 193 differentiates TCs from fibroblasts (Cismasiu et al., 2011). Therefore, it is possible that small peptides and microRNAs could also be transported.

The question then arises as to the details of these intracellular flows mediated by TCs. The gap junction syncytium could allow a free flow of electrolytes and small molecules between all connected cells including nerve endings, neural stem cells and blood vessels—either bidirectional or unidirectional depending on the structure of the gap junction. In those nervous system locations that are rich in stem cells, such as the choroid plexus/subventricular zone, the dentate gyrus and the uvea and stroma of the eye, this system might have a powerful effect on neurogenesis.

The manner of transport of these molecules along telopodes is at present unknown. The average width of a podom (100 nm) contrasts with the average width of an axon (1,000 nm). Some sections of a podom may be only 20 nm wide. TCs contain elements of the cytoskeleton such as microfilaments, microtubules and vimentin (Cretoiu et al., 2013), but it remains to be seen whether the podom contains any specialized internal transport mechanism, or whether transport depends of passive gradient-related diffusion. Also it is not clear how two-way transport could be organized along such narrow tubes. Optimal transport in terms of power and speed might be affected along such long and narrow tubes by the transport of small agents such as calcium ions. TCs contain typical Ca^2+^ release units (caveolae, sarcoplasmic reticulum and mitochondria) in the vicinity of gap junctions, which suggest calcium ions for this role (Cretoiu et al., 2012c). Synchronized voltage oscillations keyed to the release of Ca^2+^ from intracellular stores in gap-junction linked neuroepithelial cells has been reported by Yamashita (2010).

In cases where exosome transfer takes place, we have to ask where the epigenetic cargoes contained within the exosomes come from, how they are packaged in the podom and how they make their way to their targets (Smythies and Edelstein, 2013). Since exosomes are too large to navigate gap junctions, TCs must have a specific transfer mechanism to deliver exosomes to other cells. Multivesicular bodies, the parent organs of exosomes, have been described in TCs in the myometrium (Cretoiu et al., 2012d), GI tract Castiella et al. (2013) and lung (Popescu et al., 2012), and exosomes themselves have been reported in and around TCs from many tissues.

Popescu et al. (2011) state that electron tomography reveals bridging nanostructures, which connect telopodes with stem cells. Zheng et al. (2013) show examples of electron-dense connecting bridging structures between TCs and other cells including stem cells. Perhaps these nanotubes may also transport exosomes between cells.

What functions could the transfer of molecules and exosomes in the gap-junction linked TC syncytia perform? Since transport in such a system might be largely by passive diffusion, this suggests that it might exert a homeostatic function. The arrival of a large quantity of a particular agent (such as calcium ions or superoxide molecules) in any of the organelles interconnected could be counteracted by the diffusion and dilution of the superabundant agent across the system. In the other direction, a sudden drop in the level of a particular agent, for example the antioxidant glutathione, could trigger the inflow of this agent by passive diffusion from other reservoirs.

Alternatively the system could act as a series of OR gates. Molecular signals of a disturbed particular homeostatic function in either direction (for example increased inflammatory agents, antioxidants or toxic oxygen free radicals) transmitted via any of the gap junction inputs to the TC network could be carried along the network and trigger exosome release, with the resulting epigenetic modulation of the embedded stem cells. We have already drawn attention to the fact that podoms are arranged in a manner that is reminiscent of axonal boutons en passage. This flow of signals along the podom could trigger release of small signaling molecules at the gap junctions of the syncytium that would have local functional significance. It could also trigger volume release of exosomes, particularly by the podoms encountered en route, that might have a particular effect on modulating the activity of adjacent stem cells with a more distributed significance. Another question is whether, during the growth of new neurons in the adult, do the growing nerve endings export their exosome loads to the TCs, which then distribute these to neighboring neural stem cells, blood vessels and possibly astrocytes?

A third possibility is raised by the hypothesis that electrical activity within the cytoskeletal framework of neurons may carry information. Plankar et al. (2012) have recently published a comprehensive review of this topic. The cytoskeleton integrates converging signaling pathways, influences gene expression, coordinates membrane receptors and ionic flows, and localizes many cytosolic enzymes and signaling molecules. In the other direction evidence is accumulating that weak electromagnetic fields can modulate macroscopic oscillations at the network level. Coherent electromagnetic fields, produced by the longitudinal dipole oscillations in microtubules, could exert biological effects through a variety of biophysical mechanisms. These authors state “The matrix functions as a downstream target of neurotransmitters predominantly through calcium signaling, which can modify the matrix stability directly, or via signal transduction pathways by modifying phosphorylation status of binding molecules (e.g., MAP2, CaMKII), which in turn affect its structure and connectivity.”

Furthermore, Plankar et al. (2012) point out that microtubules and actin filaments carry a high net surface charge and a quantity of electrical dipoles that may enable them to carry out electrical functions related to information processing in addition to their structural roles. They list a quantity of research that has focused on long-range signal transfer within cytoskeletal elements. They point out that the intraneuronal matrix connects synapses throughout the neuron so that electrical signals originated at the postsynaptic densities could propagate throughout the system. The matrix could integrate these signals, leading to electrical modulation of voltage-gated ion channels and inducing cytoskeletal reorganization via signal transduction pathways. This raises the possibility that the extensive cytoskeletal framework in TCs might exert a similar function. They have been shown to contain cytoskeletal elements including microfilaments, microtubules and vimentin (Cretoiu et al., 2012d). In different organs TCs have been shown to possess different types of potassium, chloride and calcium channels [Rosenbaum et al. (2012); and see Cretoiu et al. (2013) for details]. In view of the sheer length and thinness of the telomeres, the transmission of any kind of chemical signal is bound to be slow. Electrical signals, possibly similar to those listed by Plankar et al. (2012), transmitted through the cytoskeleton would be practically instantaneous. Further experiments along these lines would seem to be indicated. Another mechanism that links electrophoretic phenomena and the movement of small molecules through a gap-junction linked cellular network is described by Levin (2007).

The TC network may also be involved in mechanical modulations and remodeling in various organs [see Cretoiu et al. (2012c) for a comprehensive review], but these aspects lie beyond the scope of this paper.

In conclusion, we suggest possible ways in which TCs could function as an extensive intercellular information transmission system that utilizes small molecules, exosomes—and possibly electrical events in the cytoskeleton - to modulate homeostasis, stem cell activity and other functions in many organs. This network might well be regarded as forming a very primitive nervous system at the cellular level.
